# *Plasmodium malariae* in the Colombian Amazon region: you don’t diagnose what you don’t suspect

**DOI:** 10.1186/s12936-016-1629-3

**Published:** 2016-11-29

**Authors:** Carlos Hernando Niño, Juan Ricardo Cubides, Paola Andrea Camargo-Ayala, Carlos Arturo Rodríguez-Celis, Teódulo Quiñones, Moisés Tomás Cortés-Castillo, Lizeth Sánchez-Suárez, Ricardo Sánchez, Manuel Elkin Patarroyo, Manuel Alfonso Patarroyo

**Affiliations:** 1 Molecular Biology and Immunology Department, Fundación Instituto de Inmunología de Colombia (FIDIC), Cra. 50 # 26-20, Bogotá, Colombia; 2 Gobernación del Amazonas, Calle 10 # 10-77, Leticia, Colombia; 3 School of Medicine, Universidad Nacional de Colombia, Avenida Carrera 30 # 45, Bogotá, Colombia; 4 School of Medicine and Health Sciences, Universidad del Rosario, Carrera 24#63C-69, Bogotá, Colombia

**Keywords:** Malaria, Thick blood smear, Microscopy, Nested PCR, Colombian Amazon region

## Abstract

**Background:**

Malaria is a worldwide public health problem; parasites from the genus *Plasmodium* spp. are the aetiological agent of this disease. The parasite is mainly diagnosed by microscope-based techniques. However, these have limited sensitivity. Many asymptomatic infections are sub-microscopic and can only be detected by molecular methods. This study was aimed at comparing nested PCR results to those obtained by microscope for diagnosing malaria and to present epidemiological data regarding malaria in Colombia’s Amazon department.

**Methods:**

A total of 1392 blood samples (taken by venepuncture) from symptomatic patients in Colombia’s Amazon department were analysed in parallel by thick blood smear (TBS) test and nested PCR for determining *Plasmodium* spp. infection and identifying infecting species, such as *Plasmodium vivax, Plasmodium malariae* and/or *Plasmodium falciparum*. Descriptive statistics were used for comparing the results from both tests regarding detection of the disease, typing infecting species and their prevalence in the study region. Bearing the microscope assay in mind as gold standard, PCR diagnosis performance was evaluated by statistical indicators.

**Conclusion:**

The present study revealed great differences between both diagnostic tests, as well as suggesting high *P. malariae* prevalence from a molecular perspective. This differed profoundly from previous studies in this region of Colombia, usually based on the TBS test, suggesting that diagnosis by conventional techniques could lead to underestimating the prevalence of certain *Plasmodium* spp. having high circulation in this area. The present results highlight the need for modifying state malaria surveillance schemes for more efficient strategies regarding the detection of this disease in endemic areas. The importance of PCR as a back-up test in cases of low parasitaemia or mixed infection is also highlighted.

**Electronic supplementary material:**

The online version of this article (doi:10.1186/s12936-016-1629-3) contains supplementary material, which is available to authorized users.

## Background

Malaria is a public health problem for many countries around the world. Some 3.2 billion people are at risk [1, 2] and in 2015 there were 214 million cases leading to 438,000 deaths [2]. Parasites from the genus *Plasmodium* (*Plasmodium falciparum*, *Plasmodium vivax*, *Plasmodium malariae*, *Plasmodium ovale* and *Plasmodium knowlesi*) are the aetiological agents for the disease [1, 3]. Malaria is considered to be one of the severest public health problems in Colombia as more than 90% of cases occur in 7% of all Colombia’s municipalities, rural areas (85%) being the most affected [4]. *Plasmodium vivax* represents about 70% of reported cases, whilst the rest are attributed almost exclusively to *P. falciparum* [5]. *Plasmodium malariae* infections usually do not surpass 1% [6]. Accordingly, there has not been report of cases of malaria throughout 2015, as stated by the Colombian Public Health Surveillance System’s epidemiological bulletins [7].

Microscope examination of thick blood smear (TBS) is the conventional gold standard for malaria in routine diagnosis, given its low cost and easy implementation in remote areas. Nevertheless, the amount of time spent on each sample, infrastructure maintenance, training and the ability of the personnel involved are components that heavily compromise the method’s sensitivity and the reproducibility of the results [8–10]. TBS sensitivity is 10–30 parasites per μl of blood, this being around 0.001% of infected red blood cells. However, this technique requires trained personnel, particularly when parasitaemia is low or in cases of mixed infection [11]. Molecular techniques relaying on polymerase chain reaction (PCR) and rapid diagnostic tests (RDTs) have been developed to cope with the drawbacks akin to microscopy examination. RDTs represent a cheap alternative to microscopy diagnosis. However, reports of cross-reactivity and less-than-desirable performances regarding mixed infections hinder its potential and, therefore, it has been considered inferior to microscopy in such scenarios [12, 13]. According to some studies, HRP-2 malaria RDT and microscopy have been less sensitive than PCR and especially show limited detection thresholds in situations with low parasitaemia [14–16]. Microscopy and RDTs cannot reliably detect low-density infections [17].

Conversely, PCR-based diagnostics can identify infections below the threshold of detection for microscopy and RDTs [17]. Such techniques are adaptable to individual emergency diagnosis, possess high sensitivity and specificity, and are capable of detecting low parasitaemia (about 5 parasites/μl of blood) [18, 19]. Recently, PCR has been regarded as a new gold standard for malaria diagnosis [17]. Prevalence by microscopic observation is underestimated by around 50.8% when compared to PCR [20]. Similarly, many studies show a significant share of positive infections, which have been overlooked by microscopy standard diagnostics [21–27]. Nested PCR (nPCR) shows higher sensibility than conventional and multiplex PCR diagnostics for malaria. Samples with <3000 parasites/µl of blood parasitaemia, which had positive results by the nPCR, were negative when analysed by conventional and multiplex approaches, using the same primer sets [19, 28].

A seasonal outbreak of malaria cases has been observed since 2013 in the Colombian Amazon region [29]; in 2015 such a rise was higher compared to previous years, doubling throughout 2016 [30, 31]. Problems of public order, the irregularity of malaria surveillance campaigns and Plasmodium resistance to existing anti-malarial drugs may account for this increase in malarial burden, as has been previously stated [32, 33]. Of the aforementioned factors, drug resistance is linked to accurate diagnosis, as misidentification of malaria species and degree of mixed infection inevitably lead to treatment with erroneous or incomplete medication schemes, exerting selection pressure on resistance phenotypes. This is particularly feasible for the Colombian Amazon region, a triple frontier with the Peruvian and Brazilian Amazon where the circulation of resistant *P. falciparum* and *P. vivax* phenotypes has been reported along borders [33–35].

Molecular diagnosis of a sample of symptomatic patients during the previously mentioned outbreak surprisingly revealed high prevalence values for single and mixed *P. malariae* infection according to PCR diagnostics [36], thus confirming previous suspicions that *P. malariae* prevalence may have been underestimated [22, 23, 37]. The present study represents an evaluation of microscopy observation of TBS for malaria detection and species identification, comparing this to PCR diagnosis. This work also involves the diagnosis of mixed infections and the identification of un-expected *Plasmodium* species, such as *P. malariae.* The results of this work constitute a wider and more rigorous approach towards updating the epidemiological landscape and provide a critical perspective with regard to cost-effectiveness of current diagnosis in the Colombian Amazon trapezium, an area of unstable risk and endemic transmission.

## Methods

### Study population

The samples analysed in this study came from the municipalities of Leticia (41,326 population) and Puerto Nariño (8162) in Colombia’s Amazon department (data taken from Amazonas Department Development Plan 2012–2015) [36]. The study area covered 53 settlements on the banks of the Amazon and Loretoyacu Rivers located on Colombia’s frontier with Brazil and Peru [36].

### Sample size calculation

This was a cross-sectional study. Sample size was calculated considering the estimated prevalence values from several studies performed in geographically similar populations [22, 23, 38, 39], as well as a previous work performed in the Colombian Amazon, which was regarded as a pilot survey [36]. A 1.5% prevalence was assumed as the largest sample size, taking into account all aspects to be evaluated. Accordingly, a 0.75% significance level and 95% confidence interval were chosen to avoid sample-size bias [40]. A total of 989 samples were required to fulfil the minimum sample size calculated, consistent with the information obtained when using the EPIDAT 3.1 software (Dirección Xeral de Saude Pública, Organizacion Panamericana de la Salud, Galicia, Spain).

### Sample collection

Inclusion criteria for obtaining samples from patients who were symptomatic for malaria were headache, fever during the previous 8 days, sweating, vomiting, and diarrhoea, and residing in the southern area of Colombia’s Amazon region (in and around Puerto-Nariño and Leticia). The blood samples used in this investigation were collected by personnel from the Fundación Instituto de Inmunología de Colombia (FIDIC) from July 2015 to April 2016. Each participant had a TBS test whilst blood spots on Flinders Technology Associates (FTA) cards were stored for subsequent detection of *Plasmodium* spp. by PCR.

### Ethics, consent and permissions

Each participant signed an informed consent form after having received detailed information regarding the project’s objectives, and filled in a questionnaire regarding sociodemographic characteristics; the consent form and questionnaire for minors (under 18 years old) were filled in and signed by a parent or tutor and supervised by witnesses. This project was approved by the Universidad del Rosario’s School of Medicine and Health Sciences’ research ethics committee (resolution CEI-ABN026-000161).

### Microscopy

Each TBS slide was stained with methylene blue phosphate and the cover slip was stained with 10% Giemsa (Merck, Darmstadt, Germany) for 15 min; it was then observed in immersion oil (Olympus CX21 microscope, Tokyo, Japan) for *Plasmodium* spp. parasite forms [41]. Parasite count was based on 200 leukocytes. A reference value of 8000 leukocytes was assumed for reporting parasitaemia per cu mm. A sample was considered negative when no parasite form was observed in more than 200 microscope fields observed [42]. Diagnosis was performed by personnel trained in TBS preparation, reading and reporting.

### Extracting DNA

Genomic DNA (gDNA) samples were extracted from each drop of blood collected on the FTA cards using a Pure Link Genomic DNA mini kit (Invitrogen), according to manufacturer’s specifications. The samples were eluted in a final volume of 50 µl buffer containing 10 mM Tris–HCl and 0.1 mM EDTA at pH 9.0. Extraction was verified by conventional PCR on all samples with primers directed towards a segment of the human *β*-*globin* gene to guarantee the presence of gDNA (Additional file 1: Table S1) [43]. For each reaction 1 µl of genomic DNA was used as template.

### Detecting *Plasmodium* spp. by PCR

*Plasmodium* spp. were identified by nested PCR in samples proving positive for human *β*-*globin* PCR. Specific primers for parasite 18S ribosomal subunit RNA (SSRNA) were used, following a previously described protocol with some modifications [9] (Additional file 1: Table S1). The PCR mix contained 1× buffer, 3.8 mM MgCl_2_, 1.4 mM dNTPs, 0.2 µM primers, 1 U/µl Taq polymerase (BIOLASE DNA Polymerase, Bioline), 2 µl of genomic DNA and molecular grade water (21 µl final volume). Amplification conditions were: 95 °C × 5 min, followed by 25 cycles at 94 °C × 1 min, 58 °C × 2 min and 72 °C for 2 min, with a final extension step at 72 °C × 5 min.

The corresponding PCR products were amplified again, using them as templates for a second PCR for type-specific identification of *Plasmodium* spp. (*P. falciparum, P. vivax* and *P. malariae*) using specific primers for each species (Additional file 1: Table S1). PCR mix conditions for the second PCR were: 1× buffer, 4 mM MgCl_2_, 2.5 mM dNTPs, 0.25 µM primers, 0.5 U/µl Taq polymerase and molecular grade water (20 µl final volume).

Two microlitre amplification product from the first PCR was used as template. Amplification conditions were 94 °C × 5 min, followed by 35 cycles at 94 °C × 30 s, 58 °C × 1 min and 72 °C × 4 min and a final extension cycle at 72 °C × 4 min.

gDNA samples from *P. falciparum* and *P. vivax* species were used as positive controls. Regarding *P. malariae*, a pGem-T plasmid (Promega) with the fragment of interest cloned within was used. Ultra-pure distilled water (GIBCO) was used as negative control. All products were analysed by horizontal electrophoresis (100 V, 30 min) on 2% agarose gels stained with SYBR safe (Invitrogen) and visualized on a MiniBIS Pro image analyser (DNR Bio-Imaging Systems).

### Sequencing mixed infections

Given the high prevalence found for co-infection by *Plasmodium* spp., 30 samples were randomly selected for sequencing by an ABI-3730 XL sequencer (Macrogen, Seoul, South Korea) to confirm such mixed infections.

### Statistical analysis

STATA software (Stata 12.0, Statacorp, Texas, USA) was used for obtaining descriptive statistics and determining raw values of molecular diagnosis’ performance indicators, such as sensitivity, specificity, predictive values, and related operating characteristics. Respective calculations were done regarding TBS diagnosis as a reference test. Performance indicator values have been corrected for imperfect gold standard using EPIDAT 3.1 software (Dirección Xeral de Saude Pública, Organizacion Panamericana de la Salud, Galicia, Spain), bearing previously reported sensitivity and specificity values for TBS in mind, based on other diagnostic techniques [38, 44].

## Results

### Descriptive comparison of TBS and PCR diagnosis

Prevalence values estimated by each diagnostic test were compared, according to the type of infection detected (in the case of mixed infections) and for each *Plasmodium* spp., to obtain an overall panorama of the differences between both types of diagnosis (Table 1). Table 1 shows an increase in positive frequency and estimated prevalence for all species evaluated when diagnosed by PCR; such increase was more pronounced when TBS and PCR were compared for *P. vivax* and *P. malariae*.

**Table 1:** Estimated prevalence by thick blood smear test (TBS) and PCR of 1392 samples for species and type of malarial infection

	No. of positives (prevalence)		Change in prevalence (%)^a^
	TBS n (%)	PCR n (%)	
Infective species			
*Pf*	104 (7.47)	255 (18.32)	10.85
*Pv*	509 (36.57)	996 (71.55)	34.99
*Pm*	0 (0.00)	538 (38.65)	38.65
Type of infection			
*Pv/Pf*	12 (0.86)	111 (7.97)	7.11
*Pv/Pm*	0 (0.00)	340 (24.43)	24.43
*Pf/Pm*	0 (0.00)	29 (2.08)	2.08
*Pv/Pf/Pm*	0 (0.00)	52 (3.74)	3.74

Positive sample frequencies and prevalence estimations according to each diagnostic test

TBS, thick blood smear; Pf, *Plasmodium falciparum*; Pv, *Plasmodium vivax*; Pm, *Plasmodium malariae*; co-infections are separated by a slash (/) sign

^a^Change in prevalence was calculated as the difference between prevalence values for each diagnostic test

There was also an observed increase in the frequency and prevalence estimated by PCR, regarding mixed infections; percentage change being 7.11% (*P. vivax/P. falciparum*), 24.43% (*P. vivax/P. malariae*), 2.08% (*P. falciparum/P. malariae*) and 3.74% for triple infections (Table 1). It is worth noting that TBS only detected *P. vivax/P. falciparum* mixed infections (n = 12); single, double and triple infections caused by *P. malariae* were not detected by TBS test but were so by molecular diagnosis (Table 1).

All prevalence estimations by PCR were greater than those estimated by TBS, having the highest change in prevalence the estimations regarding *P. malariae* and *P. vivax* (38.65 and 34.99%, respectively). Equally important was the change regarding mixed infections involving the detection of *P. malariae*, especially the prevalence for mixed *P. vivax/P. malariae* infection (24.43% prevalence change).

Positive and negative frequencies were then analysed for each diagnostic test, bearing in mind the frequency and percentage of positive or negative samples according to TBS and molecular diagnosis (Table 2); 43.18% (n = 601) of 1392 samples collected were positive for malaria by microscopy (TBS) whilst 56.82% (n = 791) of the samples were reported as negative by the same assay. Regarding PCR, 86.57% (n = 1205) of the samples were positive for *Plasmodium* spp.

**Table 2:** Comparing TBS and PCR diagnosis regarding malarial detection

TBS detection	PCR detection		Total, n (%)
	Positive	Negative	
Positive	596	5	601 (43.18)
Negative	609	182	791 (56.82)
Total n (%)	1205 (86.57)	187 (13.43)	1392 (100)
Agreement (%)	55.89		
Cohen’s Kappa	0.198 [0.181–0.214]		

Positive and negative sample frequency for detection by PCR diagnosis compared to TBS diagnosis

TBS, thick blood smear

Amongst the samples proving positive for TBS, 99.17% (n = 596) also proved positive by molecular diagnosis and 182 samples were negative by both tests; however, 76.99% of samples proving negative by TBS (609 samples out of 791) were positive by PCR (Table 2). The foregoing agrees with the low percentages and Cohen’s kappa calculated for both diagnostic tests, thus stressing PCR capability for detecting positive samples where TBS is not able to do so, without missing positives that the latter usually confirms. Hence, TBS-negative samples represent a huge source for possible new infections only detectable by PCR or more sensitive techniques.

The previous discrepancy between TBS negatives read as positives by PCR was explored further by analysing the frequency of single-infection, double-infection and triple-infection detected by each diagnostic test; initially by comparing the number of parasite species per infection, bearing in mind the species present and its different combinations in mixed infections.

**Fig. 1:**
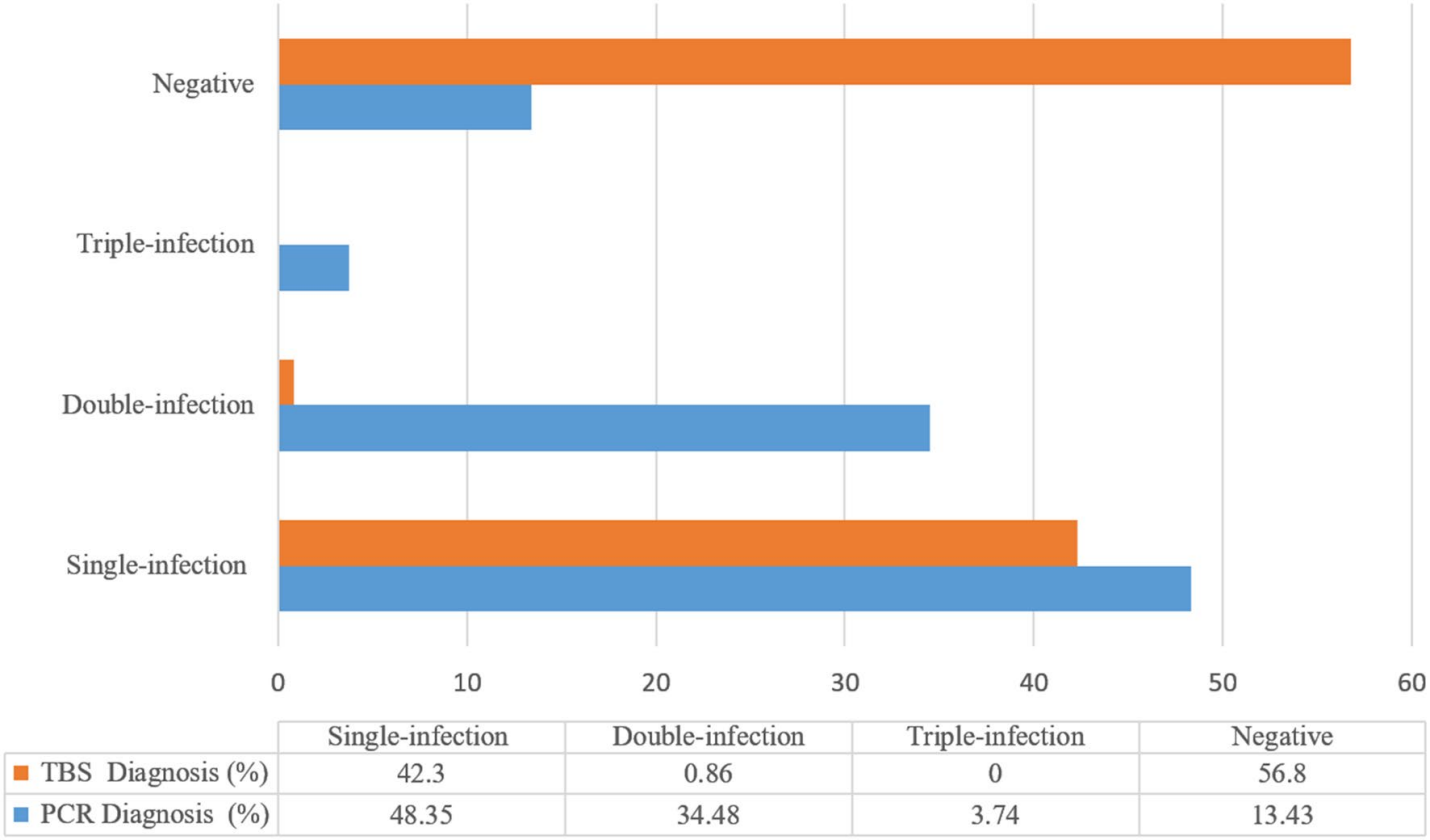
Detection agreement between TBS and PCR diagnosis with regards to single and mixed infections. Percentages of single, double and triple infections for PCR assay compared to TBS test

Figure 1 presents a parallel between TBS and PCR detection considering the number of parasite species per infection. This figure shows that TBS diagnosis only detected single-infections and double-infections. Whilst single-infection detection seems very similar to PCR results, mixed infection detection seemed impaired compared to that of the molecular diagnosis test (Fig. 1).

Considering the kind of infection classified differently by the other test, nearly 43% of single-infections by TBS were classified by molecular diagnosis as double-infections (n = 253) and only four out of 12 TBS double-infections were confirmed as such by PCR (Additional file 2: Table S2). According to Table 2, 76.99% of the 791 samples classified as negative by TBS were positive by PCR: of those 46.27% (n = 366) were single-infections, 28.19% (n = 223) double infections and 2.53% (n = 20) triple-infections (Additional file 2: Table S2). It can thus be concluded that a significant amount of single-infections and mixed infections according to the molecular diagnosis stem from samples neglected by the TBS test. Furthermore, almost half of the samples classed as single-infections by TBS were classified as mixed infections by PCR (Additional file 2: Table S2).

Table 3 shows the detection frequencies for simple and mixed types of infection, according to the species present and their combinations. This table shows the respective frequencies. TBS classed 35.7% of total participants as infection by *P. vivax* (n = 497), 6.6% by *P. falciparum* (n = 92) and 0.86% as mixed infection (*P. vivax/P. falciparum*) (n = 12). By contrast, PCR identified 35.42% individuals infected by *P. vivax* (n = 493), 4.53% by *P. falciparum* (n = 63) and 8.41% by *P. malariae* (n = 117). Three types of double infections and a triple infection were also detected by this approach: *P. vivax/P. falciparum* (n = 111), *P. vivax/P. malariae*, (n = 340), *P. falciparum/P. malariae*, (n = 29), and *P. vivax/P. falciparum/P. malariae* (n = 52). Mixed *P. vivax/P. malariae* infections according to PCR (n = 340) were mostly classified by TBS as negative (n = 138) or single-infections caused by *P. vivax* (n = 188). Regarding mixed *P. falciparum/P. malariae* infections according to PCR, 15 samples were identified as negative and 11 as single-infections, positive for *P. falciparum* by TBS. For triple-infections (n = 52), 20 samples were negative according to TBS whilst 32 samples were classified as single-infection by either *P. vivax* or *P. falciparum* (n = 20 and n = 12, respectively) (Table 3). It should be noted that *P. malariae* was detected by PCR in at least a third of the samples classified as negative by TBS. This species was present in around 40% of mixed infections (double and triple). Likewise, it is worth noting that a significant amount of mixed *P. vivax/P. malariae* infections by PCR were classified as simple infections caused by *P. vivax* by microscope; there was also the fact that 30 single-infections caused by *P. vivax* according to microscopy were identified as *P. malariae* by PCR.

**Table 3:** Comparing TBS and PCR diagnosis regarding species and types of malarial infection

Species detected by TBS	Species detected by PCR								Total n (%)
	*Pf*	*Pv*	*Pm*	*Pv/Pf*	*Pv/Pm*	*Pf/Pm*	*Pv/Pf/Pm*	Negative	
*Pf*	14	21	3	20	11	11	12	0	92 (6.6)
*Pv*	11	220	30	20	188	3	20	5	497 (35.7)
*Pv/Pf*	0	7	1	1	3	0	0	0	12 (0.86)
Negative	38	245	83	70	138	15	20	182	791 (56.8)
Total n (%)	63 (4.53)	493 (35.42)	117 (8.41)	111 (7.97)	340 (24.43)	29 (2.08)	52 (3.74)	187 (13.43)	1392 (100)

Frequencies for samples identified according to infecting species and type of mixed infection by PCR diagnosis compared to TBS assay

TBS, thick blood smear; Pf, *Plasmodium falciparum*; Pv, *Plasmodium vivax*; Pm, *Plasmodium malariae*; co-infections are separated by a slash (/) sign

### Molecular diagnosis operative performance compared to that of TBS test

Given the increase in prevalence estimated by PCR regarding TBS test and the underlying surplus of PCR positive cases explaining such discrepancies, statistical performance indicators were obtained for PCR test to confirm the apparent lower detection threshold for malaria infection.

The correspondent indicator calculation considered detecting malaria in general, detecting *P. vivax* or *P. falciparum* infection and the detection of mixed *P. vivax/P. falciparum* infection. The foregoing considered TBS as the reference standard (Table 4). Given that TBS did not detect a single sample infected by *P. malariae*, such indicators could not be obtained for simple or mixed infections involving this parasite.

**Table 4:** Statistical indicators of PCR diagnosis performance

Estimator/aspect	Sensitivity (%) [95% CI]^b^	Specificity (%) [95% CI]^b^	(PPV) (%) [95% CI]^a,b^	(NPV) (%) [95% CI]^a,b^	Accuracy (%) [95% CI]^b^	Youden index [95% CI]^b^	(AUC) [95% CI]
*Plasmodium* spp.	99.81 [99.00, 100.49]	23.79 [20.83, 26.74]	50.60 [47.51, 53.68]	99.39 [96.75, 101.65]	57.15 [54.34, 59.99]	0.24 [0.20, 0.27]	0.611 [0.596, 0.626]
*Pf*	55.82 [46.27, 65.41]	85.24 [83.21, 87.14]	26.44 [20.61, 32.48]	95.31 [93.98, 96.63]	82.69 [80.60, 84.75]	0.41 [0.31, 0.51]	0.702 [0.653, 0.751]
*Pv*	90.18 [87.55, 92.67]	44.13 [40.31, 48.06]	57.60 [53.84, 61.49]	84.23 [80.05, 88.24]	65.18 [62.12, 68.25]	0.34 [0.29, 0.39]	0.647 [0.626, 0.667]
*Pf/Pv*	8.34 [−0.17, 27.93]	92.03 [90.57, 93.45]	1.51 [−0.02, 5.04]	98.56 [97.64, 99.35]	90.82 [89.21, 92.41]	0.00 [−0.09, 0.20]	0.502 [0.42, 0.584]

Sensitivity, specificity, predictive values and performance index values for PCR assay regarding: the detection of the disease in general (any *Plasmodium* spp.), the detection of *P. falciparum* or *P. vivax* (bearing in mind samples classified as co-infection) and the detection of *P. falciparum/P. vivax* mixed infections

PPV, positive predictive value; NPV, negative predictive value; AUC, area under the ROC curve; CI, confidence interval; Pf, *Plasmodium falciparum*; Pv, *Plasmodium vivax*; co-infections are separated by a slash (/) sign

^a^Value adjusted for prevalence

^b^Value adjusted for imperfect gold standard

Regarding diagnosis for detecting malaria in general, PCR was seen to have high sensitivity (99.81%), thereby agreeing with the frequencies observed for detection in Tables 1 and 2. Consequently, relatively low specificity was observed (23.79%). Regarding the study population, positive predictive value (PPV) was 50.60% whilst negative predictive value (NPV) was notably high (99.39%). Regarding performance indexes, molecular diagnosis had above average accuracy (57.15%), together with values higher than random classification on the Youden index (0.24) and the area under the receiver operating characteristic (ROC) curve (0.611) (Table 4).

Detecting *P. falciparum* by PCR had 55.82% sensitivity and 85.24% specificity. Relatively low PPV was also observed (26.44%) whilst NPV was very high (95.31%). On the other hand, accuracy, Youden index and the area under the ROC curve (AUC) had relatively higher values regarding detection in general (Table 4).

Concerning *P. vivax* detection by PCR, very high sensitivity was observed (90.18%) together with greater specificity regarding the detection of malaria in general (44.13%). Likewise, predictive values were similar to those regarding detection in general (PPV = 57.60%, NPV = 84.23%). Regarding accuracy, the Youden index and the AUC, even though the values observed were lower regarding performance compared to *P. falciparum* detection, they were higher than those observed for the detection of malaria in general (Table 4).

Extremely low sensitivity and PPV values were observed for PCR regarding the detection of mixed *P. vivax/P. falciparum* infections; however, the highest sensitivity and NPV values were observed regarding the aspects evaluated for the diagnosis tests. In spite of high accuracy, the Youden index and AUC values suggested no better performance than that of a random discriminator regarding this aspect (Table 4).

## Discussion

For nearly 50 years in malaria-endemic areas in Colombia, diagnosis has been made by microscope observation of Giemsa-stained TBS [34]. The prevalence values given by TBS in the present outbreak agree with those reported in previous independent studies and by the Colombian Public Health Surveillance System; in such surveys *P. vivax* represented 70% of infection, whilst the remaining 30% were attributable almost exclusively to *P. falciparum* [5, 7, 36]. Likewise, *P. malariae* was regarded as sporadic, having lower than 5% prevalence [45]. Nevertheless, molecular diagnostics provided a very different epidemiological landscape, where *P. malariae* was relevant regarding both single and mixed infections. Such prevalence values agreed with what had been observed for populations from geographically related regions of the Amazon region where this diagnostic test has been used [22, 23, 36, 46]. The dramatic differences between both diagnostic tests feasibly highlighted the characteristic drawbacks of TBS: its reliance on observable parasitaemia and microscopist experience for high sensitivity and specificity, in addition to involving a risk of underestimating parasitaemia, reporting false negatives and committing errors in the identification of infecting species [15]. Consequently, such results question the usefulness of TBS when retrieving epidemiological information related to sudden outbreaks in malaria-endemic areas, despite its well-known low cost and easy implementation.

Surprisingly, nested PCR was the only diagnostic test capable of identifying *P. malariae* infection. The corresponding samples were in turn diagnosed by TBS as negative or simple infection caused by either *P. vivax* or *P. falciparum*, the predominant and regular species in the target region. Lack of quartan malaria detection by microscopy may have been related to TBS limitations per se as *P. malariae* is characterized by sustaining low infection rates and low parasitaemia [47, 48]. Similarly, the common loss of cells’ distinctive characteristics in samples treated for TBS can also account for overlooking *P. malariae* infection, given that it hampers accurate species identification [15, 22, 23, 27, 48].

*Plasmodium malariae* maximum parasite counts are usually low compared to those in patients infected with *P. falciparum* or *P. vivax* due to its longer developmental cycle (72 h for *P. malariae* versus 48 h for *P. vivax* and *P. falciparum*), lower number of merozoites produced per erythrocyte cycle, and its preference for developing in older erythrocytes; the combination of the foregoing is a trigger for the earlier development of an immune response by a human host [49].

The high share of sub-microscopic infections due to *P. malariae* reported in this work raises important questions about how individuals became infected in the first place and how long they have been bearing quartan malaria infection. The latter is relevant considering that this parasite’s blood stage persists for extremely long periods; it is often believed that it lasts for the whole life of a human host [49]. The former is important as populations in Colombia’s Amazon region co-exist with New World primates which could be a possible natural reservoir for *P. malariae* due to their striking resemblance to the zoonotic parasite *Plasmodium brasilianum* [36, 50, 51], which is now commonly thought to be an anthroponosis from *P. malariae* parasites [51].

As parasite exchange between monkeys and humans is a well documented phenomenon, the risk of primate reservoirs acting as source for outbreaks in the human population is latent. Documented chimpanzee infection with human *P. malariae* is thought to contribute to continuous parasite exchanges in Africa [52]. The preceding, combined with the characteristic low parasitaemia and long-lasting persistence of this parasite, could provide an explanation for the outbreak observed in terms of recrudescence and imported infections from nearby areas. The imported infections should be carefully considered in this particular case, taking into account that the Colombian Amazon region shares a border with both Brazil and Peru [33–35].

Many unnoticed quartan malaria parasites in mixed infections have been reported as only single infections by TBS. Such difference has usually been attributed to the fact that mixed-species infection generally implies the predominance of one species, the others having very few parasite forms [36, 53]; this gives an advantage to PCR as TBS has higher detection thresholds [54].

Erroneous identification of *P. malariae* is frequently due to haemolysis during Giemsa staining, added to morphological similarity amongst *Plasmodium* spp. during their growth stages [22, 23]. Particularly regarding *P. malariae*, this alters ring forms thus limiting routine diagnosis [55]. It is normally difficult to distinguish between *P. malariae* and *P. falciparum* parasite forms; nevertheless, in studies in South America, *P. malariae* is usually confused with *P. vivax* [22, 23, 48]. This could also account for the large amount of mixed *P. vivax/P. malariae* and *P. falciparum/P. malariae* infections which, in the present study, were rather classed as simple infections caused by just *P. vivax* or *P. falciparum* by TBS. Regardless of TBS’ inherent limitations, microscopists might have had insufficient training in recognition of *P. malariae* parasite forms. Equally, the personnel would benefit from the use of the microscopy observation of thin blood smear more extensively, given that in Colombia and other malaria-endemic regions it is used only as confirmatory analysis [22, 27]. Although thin blood smear has lower sensitivity, it better preserves the morphology of the parasite’s cells [15].

In Colombia, the prevalence of sub-microscopic infections has been observed to vary from 3 to 20%, having greater occurrence in regions where *P. vivax* is the predominant species [56]. Such a figure constitutes a worrying factor when the relationship between malaria diagnosis and treatment are taken into consideration. One possible scenario relates to favouring *Plasmodium*-resistant phenotypes due to treatment failure linked to improper diagnosis. This is particularly plausible for the *P. falciparum/P. vivax* mixed infections reported in this work, given that in Colombia, amodiaquine, followed by sulfadoxine–pyrimethamine constitute the first and second lines of treatment for falciparum malaria, respectively, whilst malaria caused by *P. vivax* is usually treated with chloroquine and primaquine schemes [34]. Therefore, underestimation of *P. vivax* infections might allow the thriving of vivax malaria phenotypes due to an incomplete elimination of liver hypnozoites, whilst underestimating *P. falciparum* infections might lead to treatment failure given its already reported resistance to chloroquine.

The present study was aimed at comparing the performance of the TBS technique to PCR diagnosis for detecting malaria in populations from the Colombia’s Amazon region. It was found that molecular diagnosis had a high sensitivity for detecting malaria in general and for malaria caused by *P. vivax*, as well as having a high NPV within the study population. These results coincided with those from previous work reporting 75–98% sensitivity for PCR regarding the identification of *Plasmodium* spp. [38, 39, 57, 58], together with 98–100% estimations for detecting *P. vivax* [38, 59, 60]. Similarly, PCR estimated higher prevalence values for the species evaluated and for certain types of co-infection, such increases having been observed in previous studies for both simple and mixed infections [22, 23, 27, 47, 48]. This result highlights PCR’s potential for confirming a clinical suspicion of malaria, in spite of being expensive and not available in health centres having limited resources [61]. This study has thus confirmed the importance of PCR-based diagnosis as the norm in future studies concerning *P. malariae* epidemiology [19, 36, 48, 53].

## Conclusion

The comparison analysed in this work highlights TBS test limitations for detecting and correctly identifying infecting species, this being related to probable low parasitaemia, as many PCR single-infections were identified as negative ones by TBS and some mixed infections were regarded as single-infections caused by ‘regular’ parasite species.

Such limitations were highlighted due to comparison with a diagnostic test having greater sensitivity (PCR), something that has previously been shown in populations of asymptomatic individuals in Colombia [62]. This study thus confirms the need for using more sensitive diagnostic techniques to enable studying epidemiological factors affecting malarial endemicity [62]. Although microscopy may continue being the gold standard for routine diagnosis and the elimination of malaria, the high incidence of asymptomatic and sub-microscopic infections highlights the urgent need for rethinking the implementation of specific strategies for monitoring and eliminating malaria from urban/peri-urban and hypo-endemic areas, the proposed target in the Colombian Public Health Plan 2012–2021 [56].

## Additional files

**Additional file 1: Table S1.** Primer sequences and amplicon sizes of the *Plasmodium* spp. identified and the *β*-*globin* gene.

**Additional file 2: Table S2.** Comparing TBS and PCR diagnosis regarding the type of infection. Frequencies according to the amount of species detected per sample by PCR assay compared to TBS test.

## Abbreviations

TBS: thick blood smear
PCR: polymerase chain reaction
PPV: positive predictive value
NPV: negative predictive value
ROC: receiver operating characteristic
AUC: area under the ROC curve
CI: confidence interval
gDNA: genomic DNA
DNA: deoxyribonucleic acid
FTA: Flinders Technology Associates
