# Supplementary material for: Plasmodium malariae in the Colombian Amazon region: you don’t diagnose what you don’t suspect
Source: Malar J. 2016 Nov 29;15:576. doi: 10.1186/s12936-016-1629-3 (PMC5129613; doi:10.1186/s12936-016-1629-3)
Supplement: Supplementary file 1 — Additional file 1: Table S1. Primer sequences and amplicon sizes of the Plasmodium spp. identified and the β-globin gene. [file 12936_2016_1629_MOESM1_ESM.doc]

**Table 1 S1. Primer sequences and amplicon sizes of the *Plasmodium* species identified and the β-globin gene, as described by Snounou *et al*. and Saiki *et al*.**

| **Primer name** | **Nucleotide sequence (5’- 3’)** | **Parasite targeted** | **Amplicon length (bp)** |
| --- | --- | --- | --- |
| rPLU 5 | CCTGTTGTTGCCTTAAACTTC | *Plasmodium sp*. (first amplification) | ~1200 |
| rPLU6 | TTAAAATTGTTGCAGTTAAAACG |
| rFAL 1 | TTAAACTGGTTTGGGAAAACCAAATATATT | *P. falciparum* | 205 |
| rFAL2 | ACACAATGAACTCAATCATGACTACCCGTC |
| rVIV 1 | CGCTTCTAGCTTAATCCACATAACTGATAC | *P. vivax* | 117 |
| rVIV2 | ACTTCCAAGCCGAAGCAAAGAAAGTCCTTA |
| rMAL1 | ATAACATAGTTGTACGTTAAGAATAACCGC | *P. malariae* | 144 |
| rMAL2 | AAAATTCCCATGCATAAAAAATTATACAAA |
| GH20 (forward) | GAAGAGCCAAGGACAGGTAC | Human β-globin locus | 268 |
| PCO4 (reverse) | CAACTTCATCCACGTTCACC |
