# Supplementary material for: Plasmodium malariae in the Colombian Amazon region: you don’t diagnose what you don’t suspect
Source: Malar J. 2016 Nov 29;15:576. doi: 10.1186/s12936-016-1629-3 (PMC5129613; doi:10.1186/s12936-016-1629-3)
Supplement: Supplementary file 2 — Additional file 2: Table S2. Comparing TBS and PCR diagnosis regarding the type of infection. Frequencies according to the amount of species detected per sample by PCR assay compared to TBS test. [file 12936_2016_1629_MOESM2_ESM.docx]

**Table S2: Comparing TBS and PCR diagnosis regarding the type of infection.** Frequencies according to the amount of species detected per sample by PCR assay compared to TBS test.

| Type of infection by TBS | Type of infection by PCR | | | | Total n (%) |
| --- | --- | --- | --- | --- | --- |
|  | *Single-infection* | *Double-infection* | *Triple-infection* | *Negative* |  |
| *Single-infection* | 299 | 253 | 32 | 5 | 589 (42.3) |
| *Double-infection* | 8 | 4 | 0 | 0 | 12 (0.86) |
| *Negative* | 366 | 223 | 20 | 182 | 791 (56.8) |
| Total n (%) | 673 (48.35) | 480 (34.48) | 52 (3.74) | 187 (13.43) | 1,392 (100) |

TBS= *thick blood smear*
